# The 4^th^ NextGen therapies for SJIA and MAS: part 3 clinical trials in refractory SJIA: historic controls as an alternative to a withdrawal design study

**DOI:** 10.1186/s12969-023-00866-z

**Published:** 2024-01-03

**Authors:** Fabrizio de Benedetti, Alexei A. Grom, Hermine Brunner

**Affiliations:** 1 Division of Rheumatology, Ospedale Pediatrico Bambino Gesù, Rome, Italy; 2 Division of Rheumatology, Cincinnati Children’s Hospital Medical Center, University of Cincinnati, Cincinnati, OH USA

**Keywords:** Refractory SJIA, Refractory SJIA trial design, Historical controls, MAS

## Abstract

The substantial morbidity and mortality associated with refractory systemic JIA underlies the need for new treatment approaches. However, progress in this area has been limited by the difficulty of enrolling these patients in clinical trials with traditional designs, particularly in patients presenting with the life-threatening macrophage activation syndrome. At the NextGen 2022 conference, there was group consensus that using historical cohorts as a control group to avoid the need for a placebo-arm or drug withdrawal was highly desirable and might be acceptable for clinical trials in MAS to support medication efficacy and safety. However, if historic controls were used in a trial, it would be important to ensure that the historic cohort matches the study group in terms of clinical characteristics (such as disease severity and exposure to other medications), and that disease outcome in both groups is assessed using the same outcome measures. The discussions at the NextGen 2022 conference focused on the potential strategies to achieve these goals.

## Introduction

The substantial morbidity and mortality associated with refractory systemic JIA underlies the need for new treatment approaches. However, progress in this area has been limited by difficulty of enrolling these patients in clinical trials with traditional designs. As a fundamental principle, the data generated in a trial should demonstrate efficacy of new products, and in general, there is a need for a control group to show that the efficacy is not due to background medications and that safety of the new product is appropriate. Traditionally, placebo controlled parallel, or withdrawal designs have been used to assess safety and efficacy, and new medications were only started after wash-out of concurrent advanced medications. However, the severity of refractory SJIA, particularly when it is associated with MAS or the lung disease, high risk for rapid life-threatening deterioration, and often large numbers of background medications, make traditional trial designs difficult to apply to this patient population. The goal of this session was to discuss potential alternative trial strategies and designs that would be acceptable to patients, clinicians, and regulatory agencies.

## Historic controls as an alternative to a placebo-controlled design study

The discussion started with parents’ perspective on this issue. Although there was consensus among parents that a randomized withdrawal design would indeed provide a greater level of evidence, and therefore, higher chance of study results providing support of medication efficacy and safety by regulatory agencies, the majority of the parents had major reservations against enrolling their children into this type of studies. Parents’ stories presented during this session clearly supported that some of these patients decompensate rapidly. Thus, placebo exposure or medication withdrawal seemed to be unethical; a notion supported by medical providers. There was also a suspicion that some of these patients might not regain the same degree of disease control that the new medication was withheld as is the case in patients randomized to the placebo arm of a withdrawal design study. However, some clinicians and parents indicated that a withdrawal design could be acceptable to them, if even a minor flare would trigger a restart of study medications during the double-blinded withdrawal part of a trial. In this scenario, even a slight worsening would allow to re-start treatment immediately, thus decreasing the risk of letting the patient decompensate. Most of the discussion, however, was spent on using historical cohorts as a control group thus avoiding using blinded studies, including withdrawal design trials, at all. Clearly, open-label study design where all patients receive promptly active treatment (access to new study medication) was overwhelmingly preferred, given the aforementioned concerns of unnecessary flares and potentially irreversible damage acquisition from each flare.

There are many examples of successful use of historic control cohorts, mainly in trials in monogenic diseases that would typically have a more consistent and better-defined disease course as opposed to the patients with refractory SJIA where the natural evolution of its complications still needs to be defined better. FDA representatives felt more comfortable approving medications using historic controls for diseases with well-defined pathways, i.e. monogenic diseases [1–4], as opposed to diseases with not-fully understood mechanisms of disease, as is the case in refractory SJIA.

### Outcome assessment in historic cohorts

If historic controls were used, it would be important to ensure that the historic cohort matches the study group in terms of clinical characteristics (such as disease severity and exposure to other medications), and disease course in both groups is assessed using the same outcome measures. Based on the earlier FDA presentation, it was imperative that the outcome measures used in the trial are clinically significant, and objective. Improvement in the typical laboratory parameters of MAS as well as the ability to reduce the exposure to corticosteroids were proposed as potential outcome measures by several clinicians. From the FDA perspective, however, laboratory criteria were considered surrogate outcomes and do not suffice to support medication efficacy alone. The same was true for the ability to reduce the dose of corticosteroids. Another point was made, that for refractory SJIA, given the urgent need for new treatments, a prospective study that would take many years to complete would not be acceptable to the parents. Therefore, the data from historic control cohorts would need to be collected retrospectively. Therefore, the next part of the discussion was focused on potential clinically significant outcome measures that could be captured retrospectively in patients with acute MAS as a primary outcome measure. Potential outcome measures mentioned and deemed relevant by many meeting participants were:

- Length of ICU stay
- Length of hospitalizations
- Duration of fever
- Need for ventilation,
- Death
- Reduction of number for hospitalizations long term.

One caveat brought up by parents was that criteria for the need of hospitalization changed during the epidemics of COVID. A proposed solution was to try to capture and count outpatient infusions and IV pulse steroids.

The ongoing open-label trial of emapalumab in MAS/SJIA was brought up as an example [5]. The main Efficacy Outcome in this trial was: “MAS Remission at Week 8”, where the MAS remission criteria is defined in Table 1 below:

**Table 1:** MAS Remission Criteria

^MAS Remission Criteria:^
✓ Resolution of MAS clinical signs and symptoms present at baseline:
∙ MAS was considered resolved if VAS ≤1/10
And
✓ Normalization of laboratory parameters relevant to MAS:
∙ WBC >LLN
∙ Platelet count >LLN
∙ LDH <1.5 ULN
∙ ALT <1.5 ULN
∙ AST <1.5 ULN
∙ Fibrinogen >100 mg/dL
∙ Ferritin ≤80% from values at screening or baseline (whichever is higher) or <2000 ng/mL, whichever is lower

All the participants agreed that, perhaps apart from the VAS of MAS severity (see table above), all other of these clinical and laboratory parameters could be collected retrospectively. The meeting participants were of the opinion that this would be a reasonable path to generate a contemporary historic control. The consensus was that this retrospective registry should be very inclusive with broad inclusion criteria to include MAS patients representing the full spectrum of this disease [6]. To create a representative control cohort for a specific clinical trial, filtering the registry data using the trial inclusion criteria would allow selecting a subgroup of MAS patients whose clinical characteristic would match those in the trial.

Another important consideration discussed was that there were some geographic differences in the prevalence and severity of MAS and refractory SJIA. Further, the introduction of IL-1 and IL-6 inhibiting biologics in 2012 and, more recently JAK-inhibitors have influenced the phenotypes and clinical presentation of MAS. There was consensus that the registry should focus on MAS patients diagnosed after 2012 and include patients from geographical areas that would reflect enrollment sites in the trials.

### Retrospective historical cohorts as a control arm for trails in SJIA associated lung disease

The group then discussed potential outcomes measures for the patients with SJIA associated lung disease with the focus on the lung disease itself. The group proposed to following measures:

1. High resolution chest CT
  There are at least 6 distinct radiographic features characteristic of the SJIA-LD: subpleural thickening, inter-septal thickening, tree-and-bud changes, ground-glass opacities, areas of consolidation, and mediastinal LAD [7, 8]. HRCTs could be all re-read by the same highly experienced radiologists; and each imaging feature could be assigned a score based on severity leading to a composite score that would reflect the overall lung disease activity. The scoring system would certainly need to be validated, but the main advantage of this approach is that it could be achieved retrospectively. One limitation of HRCT is that it fails to distinguish well between the inflammatory and fibrotic components of the lung disease. The treatment may provide a full control of inflammation in the lungs, but the fibrotic component may progress. Therefore, HRCT alone may not be sensitive enough to measure true improvement. There was group consensus that HRCT imaging as a SJIA-ILD outcomes should be supplemented by functional pulmonary assessments.
2. Pulmonary function testing
  The case presented by Dr. Ed Behrens clearly showed that the combination of HRCT and PFTs might be a very reliable marker of lung disease activity. The functional assessment may include:
  (1) O_2_ requirement, a readily measurable objective outcome that is regularly recorded for patients with pulmonary involvement and can be extracted from the EMR retrospectively. (2) 6-min walk test, a functional measure with high sensitivity to change to be collected prospectively. These data are unlikely to be available in the EMR for retrospective data capture. (3) Overnight sleep oximetry, another sensitive, objective and responsive outcome measure for a prospective study, but the data may not be available retrospectively and cannot be collected from patients requiring ventilator support.

## Summary of discussions

In summary, there was group consensus that using historical cohorts as a control group to avoid the need for a placebo-arm or drug withdrawal was highly desirable and might be acceptable for clinical trials in MAS to support medication efficacy and safety. However, if historic controls were used in a trial, it would be important to ensure that the historic cohort matches the study group in terms of clinical characteristics (such as disease severity and exposure to other medications), and that disease outcome in both groups is assessed using the same outcome measures. Given the urgent need for new treatments, a prospective study to establish a control cohort that would take many years to complete would not be acceptable to the parents. Therefore, the data from historic control cohorts would need to be collected using EMR review. The consensus was that this retrospective registry should be very inclusive with broad inclusion criteria to include MAS patients representing the full spectrum of this disease. Another important consideration is that the introduction of IL-1 and IL-6 inhibiting biologics in 2012 and, more recently JAK-inhibitors, has made an impact on the clinical presentation of MAS; therefore, the registry should focus on MAS patients diagnosed after 2012.

## Data Availability

All the data discussed during the meeting have now been published and appropriately referenced at the end of the manuscript.

## Abbreviations

ALT: Alanine transaminase
AST: Aspartate aminotransferase
CT: Computerized tomography
EMR: Electronic medical records
HRCT: High-Resolution Computed Tomography
ICU: Intensive Care Unit
IL: Interleukin
ILD: Interstitial lung disease
IV: Intravenous
JAK: Janus kinase
JIA: Juvenile Idiopathic Arthritis
LLN: Lower limit of normal
LD: Lung Disease
LAD: Lymphadenopathy
MAS: Macrophage activation syndrome
PFTs: Pulmonary Function Tests
SJIA: Systemic Juvenile Idiopathic Arthritis
ULN: Upper limit of normal
FDA: U.S. Food and Drug Administration
WBC: White blood cells
VAS: Visual Analogue Scale
